# Effectiveness of a Brief Educational Video on the Correct Placement of Automated Chest Compression Devices: A Prospective Study

**DOI:** 10.7759/cureus.99629

**Published:** 2025-12-19

**Authors:** Garrett Jordan, Keith Baker, Kevin Kover, Bryan McCrea, Bryan R Wilson, Rebecca Jeanmonod

**Affiliations:** 1 Emergency Medicine, St. Luke's University Health Network, Bethlehem, USA; 2 Medical Toxicology, St. Luke's University Health Network, Bethlehem, USA; 3 Emergency Medical Services Fellowship, St. Luke's University Health Network, Bethlehem, USA; 4 Department of Emergency Medicine, St. Luke's University Health Network, Bethlehem, USA

**Keywords:** cardiac arrest, cpr, mechanical cpr, quality improvement projects, resuscitation

## Abstract

Objective

The time to the onset of high-quality cardiopulmonary resuscitation (CPR) is directly correlated with the chance of survival for cardiac arrest. The use of automated chest compression devices, such as the LUCAS, has become a common means of performing CPR. We sought to determine whether an educational intervention improves the correct placement of an automated chest compression device, and to identify whether placement accuracy varies by performer profession or years of experience.

Methods

Emergency department technicians, nurses, residents, and attending physicians were recruited to perform placement of a LUCAS device before and after watching an educational video created by the LUCAS manufacturer. The correct placement of an automated chest compression device was determined by three different emergency medicine attending physicians, using manufacturer instructions. The distance between the volunteer’s placement of the LUCAS and the ideal placement location, before and after watching the educational video, was recorded. The Wilcoxon rank-sum test was used to compare the pre- and post-education measurements.

Results

A total of 50 employees participated in the study. The median distance from correct placement was 3.2 cm prior to the educational intervention and 3.3 cm after the educational intervention, indicating no significant change. There was no difference in the accuracy of device placement when comparing physicians and non-physicians. There was no relationship between years of experience and the accuracy of device placement. All participants found participating in the educational intervention useful.

Conclusion

In this small, single-system, prospective study, we found that a single, brief, passive educational intervention does not improve the correct placement of an automated chest compression device. Additional data are needed to understand how this video might be utilized in an ongoing quality improvement project or as part of a larger, integrated educational intervention study.

## Introduction

Background

Cardiopulmonary resuscitation (CPR) is a lifesaving procedure performed when the heart stops beating. It keeps blood flow active and extends the opportunity for successful resuscitation. Nearly 350,000 Americans die from heart disease every year, and immediate CPR has been shown to double, or even triple, the chances of survival after cardiac arrest [1]. Sudden cardiac arrest is a leading cause of death in much of the world, and cardiac disease is the leading cause of death in the United States [2]. Because of this, it is imperative that timely, effective CPR be delivered to those suffering from cardiac arrest.

Despite the proven benefits of prompt, high-quality CPR, the quality of performance of this vital procedure is variable and often below recommended standards. Abella et al. showed that compression rates were too slow 28.1% of the time, and compression depth was too shallow 37.4% of the time [3]. Quality of CPR directly impacts outcomes. Inadequate depth of chest compression reduces survival-to-discharge rates by 30%, and compressing too slowly reduces return of spontaneous circulation (ROSC) from 72% to 42% [4]. It has been shown that even among trained professionals, chest compressions are not given nearly half the time without spontaneous circulation [5]. In 2015, Talikowska et al. demonstrated that each of these individual parameters was associated with changes in outcomes, with greater adherence to recommendations associated with improved outcomes [6].

For these reasons, the use of automated chest compression devices has become an increasingly common means of CPR. These devices are often used as an alternative to manual CPR. They provide several potential advantages over manual CPR, including a fixed rate and consistent depth of compression. Furthermore, they eliminate the factor of performer fatigue and can free up additional personnel to assist in the resuscitation. Manufacturers of these devices boast studies that have demonstrated statistically significant increases in ROSC with the use of these devices [7]. However, other well-performed randomized controlled trials have not demonstrated the same benefit. The COMPRESS-RCT is one of the best examples of this. This study randomized patients to mechanical versus conventional CPR and found no difference in either ROSC, survival to hospital discharge, or survival with a good neurological outcome [8]. 

Standardized CPR training courses, such as Basic Life Support (BLS) and Advanced Cardiovascular Life Support (ACLS), primarily emphasize manual CPR, ensuring a consistent level of competence among certified providers. However, these courses do not include instruction on automated chest compression devices. This gap in training introduces variability in provider proficiency with these devices. Given the critical role of high-quality CPR in patient outcomes, the ability to correctly operate an automated CPR device should be considered a lifesaving skill. Improper use, particularly incorrect placement, can result in inadequate compressions and potential patient harm. A recent meta-analysis reported that mechanical CPR is associated with a significantly higher incidence of compression-related injuries (OR 1.29), including rib fractures (OR 1.23), multiple rib fractures (≥3) (OR 1.45), heart lesions (OR 2.10), and liver lesions (OR 2.75) [9]. To date, no studies have evaluated whether educational interventions improve the correct placement of automated chest compression devices. We hypothesize that such training would enhance placement accuracy.

Objective

We sought to determine whether a brief educational intervention improves the correct placement of an automated chest compression device, and to identify whether variables, such as profession or years of experience, play a role in placement accuracy.

## Materials and methods

Study design

This was a single-blinded, interventional study evaluating 50 Emergency Department (ED) employees’ placement of the LUCAS device before and after a brief educational intervention that consisted of a short video from the manufacturer. This study was performed from February 2024 to March 2024 at two different hospital EDs. Of these employees, 35 were resident or attending physicians, and 15 were nurses or techs. This study was reviewed by our institution’s IRB and was found to be exempt.

 Protocol

The study leaders agreed upon the correct placement of the automated chest compression device (LUCAS 3 Chest Compression System) and used an invisible UV marker to mark the correct location for LUCAS device placement on the study manikin (“SimMan,” Laerdal Medical, Wappingers Falls, NY, USA). Study leaders watched a manufacturer-developed educational video for in-hospital use [10] and viewed diagrams [11] on the LUCAS website to determine the correct location of the device. The educational video viewed by the study leaders would later be viewed by the participants. The video content walked the viewer step by step through the appropriate setup and use of the LUCAS device. Leaders used exact measurements with a ruler to ensure that the device was centered between the manikin nipples and at the correct height above the manikin xiphoid process. Participants were voluntarily recruited and self-reported their profession and years of experience. Participants were then observed placing the automated chest compression device prior to viewing the educational video. Measurements were taken between the participants’ placement of the device and the correct location, using a black light to reveal the correct location for the LUCAS device. The measurements were obtained by measuring the point of maximal distance from the edge of the suction cup nearest the correct placement to the far edge of the blacklight marker. Participants were unaware of these measurements. They subsequently completed training via the educational video provided by the LUCAS manufacturer’s website. After watching the video, participants placed the device again. Post-education measurements were recorded independently in a spreadsheet (Microsoft Excel®, Redmond, WA, USA) for review by a third party. Finally, participants completed a survey regarding the subjective utility of the educational video. Participants were not aware of what was being studied, nor were they present in the room when this measurement was being taken. Following the training, participants answered a survey on whether they felt that participating in the educational intervention was useful.

Outcome measures

The primary outcome was to determine whether an educational intervention can improve the correct placement of an automated chest compression device, as measured by the distance of maximal deviation of the device’s suction cup from the pre-determined correct location on the center of the chest. Secondary outcomes included: (1) whether pre-education and post-education placement accuracy varied by performer profession (resident or attending physician, or nurse/ED technician) or years of experience, and (2) whether employees found participating in an educational intervention useful.

Statistical analysis

Data were analyzed by an independent third party with expertise in statistics. Measurements were compared using a Wilcoxon rank-sum test. Descriptive statistics were calculated for the additional data points, and statistical analysis was performed using a Mann-Whitney test.

## Results

**Figure 1:**
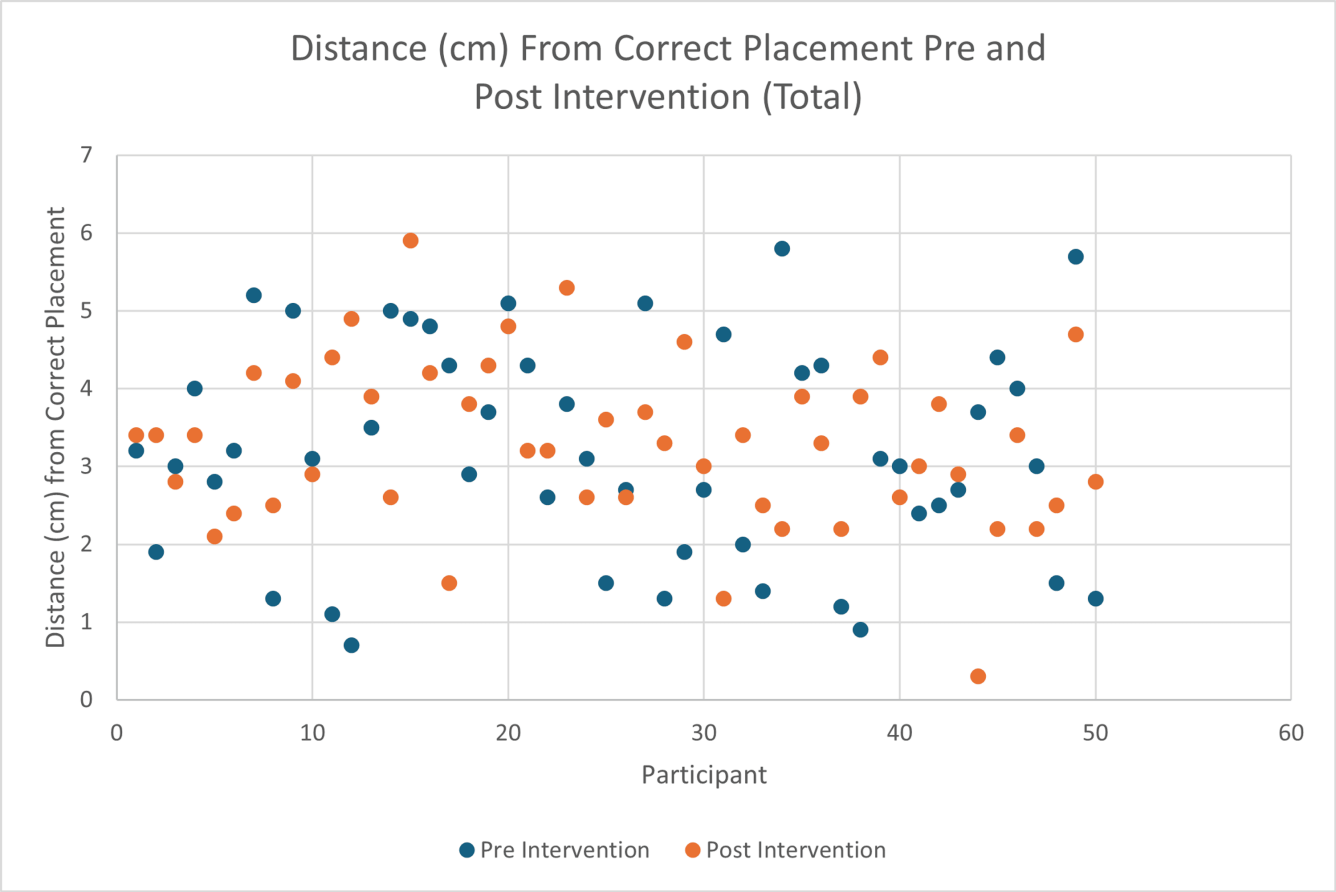
Average distance from correct placement before and after viewing the LUCAS educational video provided by the manufacturer.

Of the 50 included employees, 35 were resident or attending physicians, and 15 were nurses or ED technicians. The average distance from correct placement was 3.2 cm (IQR 2.0-4.3) prior to the educational intervention, and 3.3 cm (IQR 2.6-3.9) after the educational intervention (p = 0.56). Of the 50 employees, 25 improved their placement, and 25 worsened their placement after the educational intervention (Figure 1).

**Figure 2:**
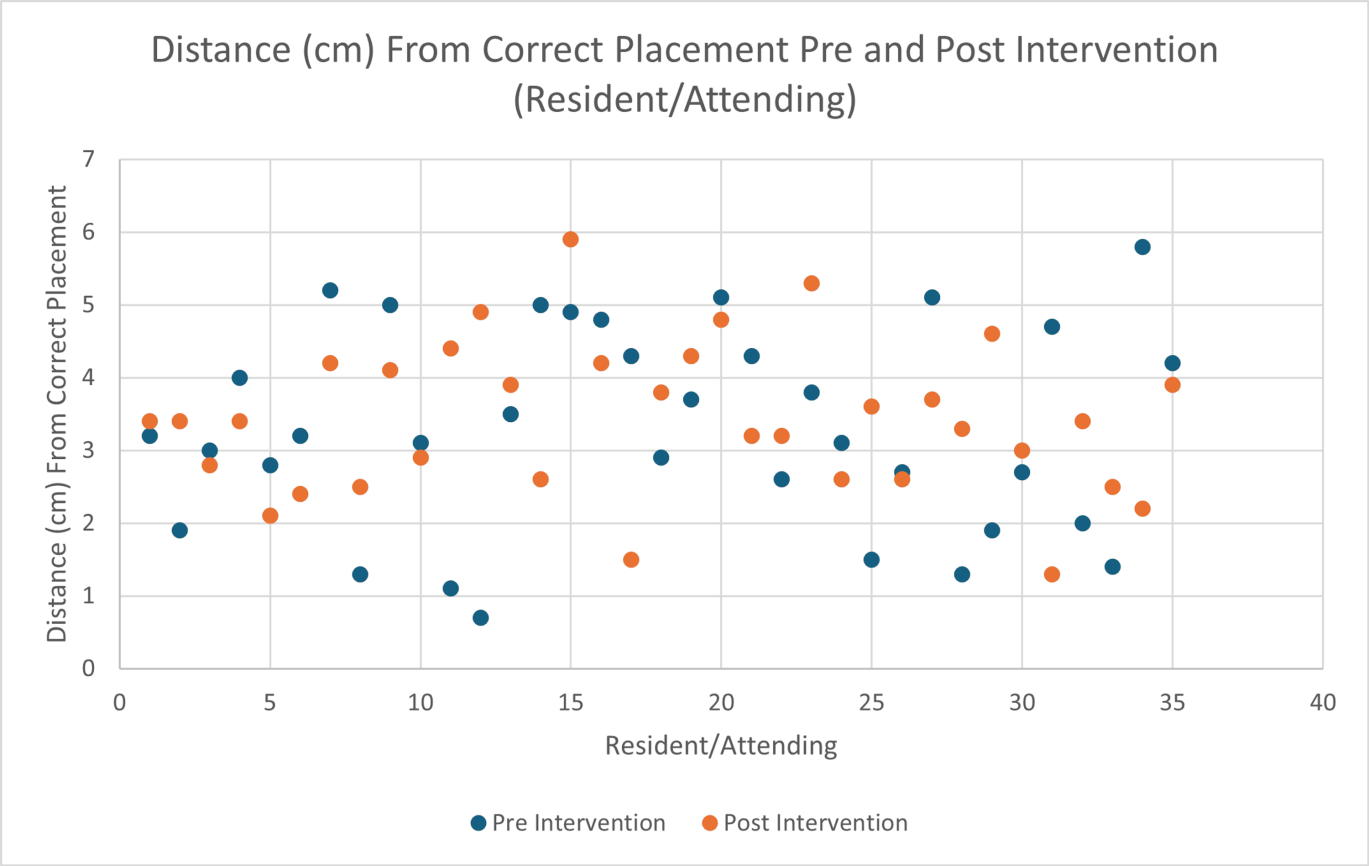
Average distance from correct placement before and after viewing the LUCAS educational video provided by the manufacturer specifically for resident physicians and attending physicians.

When examining the differences between residents and attending physicians, no significant difference was noted (3.31 cm pre-education and 3.43 cm post-education, p = 0.6916) (Figure 2).

**Figure 3:**
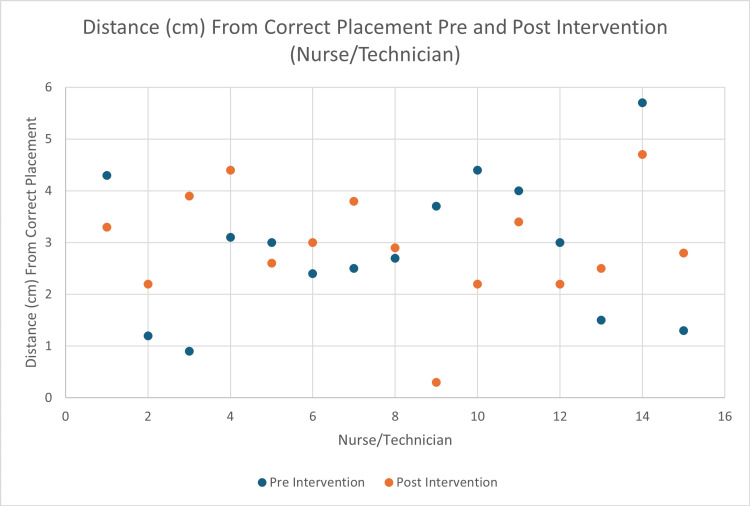
Average distance from correct placement before and after viewing the LUCAS educational video provided by the manufacturer specifically for registered nurses and emergency room technicians.

Similarly, no significant difference was noted between nurses and ED technicians (2.91 cm pre-education and 2.94 cm post-education, p = 0.9411) (Figure 3).

A total of 18 residents/attendings improved their placement after the educational intervention, and 17 residents/attendings worsened. A total of seven nurses/techs improved their placement after the educational intervention, and eight nurses/techs worsened. There was no difference in the accuracy of device placement when comparing physicians (n = 35) and non-physicians (n = 15) (p = 0.30, Mann-Whitney).

**Figure 4:**
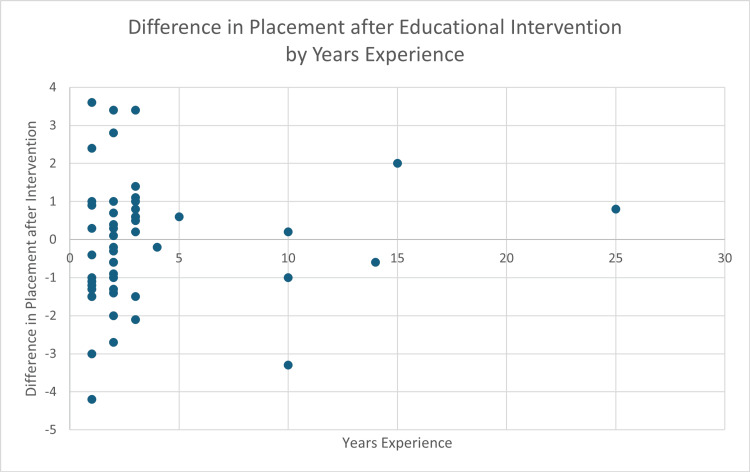
Average distance from correct placement after viewing the LUCAS educational video provided by the manufacturer specifically based on years of experience for all participants.

The resident/attending group had fewer average years of experience (2.43 years) compared to the nurse/tech group (6 years). Although the nurse/tech group had better baseline placement, years of experience within groups did not have an impact on either initial placement or change in placement following the educational intervention (pre-intervention R² = 0.007; post-intervention R² = 0.05) (Figure 4). All the employees found the educational intervention to be useful, based on the post-study survey.

## Discussion

We conducted a prospective study including employees at two large EDs in a single hospital network in Pennsylvania to determine whether an educational intervention can improve the correct placement of an automated chest compression device. Though study subjects found the intervention helpful, we found that the use of a brief educational intervention in isolation does not improve the correct placement of an automated chest compression device. This is not surprising, as a single, isolated, passive intervention is unlikely to result in lasting change in a system, emphasizing the need for ongoing, multifaceted, comprehensive training on automated chest compression device use and application.

The presence of automated chest compression devices is increasing in use among EDs and in the prehospital setting [12]. Currently, while BLS and ACLS provide a national standard of training for CPR, there is not a comparable certification in mechanical CPR. This discrepancy could potentially allow for provider variability due to a lack of experience with the LUCAS device. Our study suggests that video education does not help with device placement. Interestingly, nurses and techs, who are more likely to be the ones applying these devices, had a trend toward better baseline placement than residents and attendings. This may indicate that experience plays a role in appropriate placement.

This study should be interpreted in the context of its limitations. First, it was entirely performed by in-hospital providers. Automated chest compression devices are often applied en route by prehospital emergency medical services (EMS) clinicians. With nursing/techs - seemingly more experienced with device application - having a trend toward better placement, it is possible that EMS providers would be even better at device application. Second, a manikin was used to determine the correct placement. While the manikin is lifelike and anatomically accurate, certain intangibles may make placing a device on a human easier, such as the degree of tactile feedback from palpating real landmarks. Only 50 participants were recorded in this study, although it is likely that these results would be replicated on a larger scale. The results came from a single hospital network, limited to ED staff. It is unclear how the results would compare if the study were repeated to include other hospital networks and departments outside of the ED.

Lastly, this study did not compare measurements of proper placement of the LUCAS device to manual CPR. It is possible that similar results for erroneous hand placement would be found if the study compared ideal versus actual hand placement during CPR. If hand placement were found to be more accurate than the correct placement of an automated chest compression device, it would show a distinct advantage of manual CPR. 

## Conclusions

In this small, single-system prospective study, we found that a single, brief, passive educational intervention does not improve the correct placement of an automated chest compression device. Additional data are needed to understand how this video might be utilized in an ongoing quality improvement project or as part of a larger, integrated educational intervention study.
