# Correction: Effectiveness of a Brief Educational Video on the Correct Placement of Automated Chest Compression Devices: A Prospective Study

**DOI:** 10.7759/cureus.c395

**Published:** 2026-01-15

**Authors:** Garrett Jordan, Keith Baker, Kevin Kover, Bryan McCrea, Bryan R Wilson, Rebecca Jeanmonod

**Affiliations:** 1 Emergency Medicine, St. Luke's University Health Network, Bethlehem, USA; 2 Medical Toxicology, St. Luke's University Health Network, Bethlehem, USA; 3 Emergency Medical Services Fellowship, St. Luke's University Health Network, Bethlehem, USA; 4 Department of Emergency Medicine, St. Luke's University Health Network, Bethlehem, USA

This article has been corrected to add Rebecca Jeanmonod as the final author.

